# A novel *ABO* splice site variant underlying the A_3_ phenotype: immunogenetic basis and functional dissection

**DOI:** 10.3389/fgene.2026.1839848

**Published:** 2026-06-19

**Authors:** Hong-Li Guan, Jue Hou, Jia-Liang Gao, Jian Li, Xue-Mei Zhang, Xue Chen, Xue-Mei Fu

**Affiliations:** Blood Grouping Reference Laboratory, Chengdu Blood Center, Chengdu, Sichuan, China

**Keywords:** A3 phenotype, ABO, immunogenetic, splice site variant, transfusion

## Abstract

**Background:**

Accurate ABO blood group typing is essential for transfusion safety. However, ABO subtypes resulting from genetic variation can complicate this process. In contrast to the extensively studied exonic variants in the *ABO* gene, splice-site variants are rarely reported, and the mechanisms underlying their contribution to ABO subtypes remain poorly characterized.

**Methods:**

Four unrelated, healthy blood donors exhibiting mixed-field agglutination with anti-A reagents were subjected to Sanger sequencing. Long-range PCR coupled with nanopore sequencing was further performed to resolve the phased haplotype across the entire *ABO* locus. The impact of the identified variant on splicing was predicted *in silico* and validated by an *in vitro* minigene assay; its effects on protein structure were assessed using bioinformatic tools. Short tandem repeat (STR) analysis was also conducted to exclude blood group chimerism.

**Results:**

Four unrelated Chinese blood donors exhibiting mixed-field agglutination with anti-A reagents were identified. Sanger sequencing and nanopore sequencing revealed that all four probands harbored a novel c.239 + 6T>C variant in the ABO*A1.01 allele, with short tandem repeat (STR) analysis ruling out chimerism as the cause of the mixed-field agglutination. *In silico* analysis predicted activation of a cryptic splice site, leading to retention of a 55-bp intronic fragment in the mRNA transcript. Further, the minigene splicing assay validated this predicted aberrant splicing pattern and additionally revealed trace production of the normally spliced transcript. Secondary and three-dimensional structural modeling further predicted that the resulting aberrant transcript encodes a severely truncated and catalytically compromised glycosyltransferase A, providing a molecular basis for the observed A antigen expression defect.

**Conclusion:**

In conclusion, a novel ABO variant (c.239 + 6T>C) responsible for the A_3_ phenotype was identified, and the mechanism underlying this phenotype was elucidated. By elucidating how a splice site variant disrupts glycosyltransferase function and alters antigen expression, this study contributes to a deeper understanding of the immunogenetic basis of ABO variation, with implications for transfusion safety and personalized immunohematology.

## Introduction

Since its discovery in 1900, the ABO blood group system has played an irreplaceable role in transfusion medicine and organ transplantation (Wang et al., 2022; Hu and Zhang, 2026). The ABO gene, located on chromosome 9 (9q34.2), encodes glycosyltransferases A (GTA) and B (GTB), which determine ABO phenotypes by catalyzing the addition of specific sugar residues to the H antigen acceptor substrate on red blood cells, resulting in the formation of A and B antigens, respectively (Paulson and Colley, 1989). Variations in this gene can alter the activity or specificity of these enzymes, giving rise to ABO blood group subtypes. Among these, the A_3_ phenotype is serologically defined by a characteristic mixed-field agglutination pattern with anti-A reagents, manifested as coexisting agglutinated red cell clumps and free red cells (Barjas-Castro et al., 2000). This mixed-field agglutination arises from *ABO* gene variations present in a homogeneous genetic background, instead of from chimerism, which involves the coexistence of two genetically distinct cell populations (Yamamoto et al., 1993). To date, at least 13 A_3_ alleles have been identified, harboring variations in exonic regions, intronic regulatory elements, or splice-site regions (Red blood histo). While previous molecular studies have largely focused on exonic and intronic regulatory variants (Li et al., 2007; Svensson et al., 2005; Thun et al., 2024), the role of splice-site variations in the etiology of the A_3_ phenotype remains poorly understood, despite their well-documented involvement in other weak subgroups such as A_el_, and B_3_ (Yu et al., 2002; Chen et al., 2015). Thus, the present study aimed to address this gap by characterizing the functional impact of the c.239 + 6T>C variant in A_3_.

Faithful pre-mRNA splicing relies on accurate recognition of exon-intron boundaries. Splice-site variants can disrupt splicing through multiple mechanisms, including compromising canonical splice sites, activating cryptic splice sites, or altering the function of splicing regulatory elements. These disruptions can lead to exon skipping, intron retention, or the generation of aberrant splice variants, ultimately producing abnormal mRNA transcripts (Wilkinson et al., 2020; Xie et al., 2025; Anna and Monika, 2018). Such transcripts often harbor frameshifts, premature termination codons, or deletions of critical functional domains, ultimately yielding non-functional or severely impaired enzymatic proteins that cannot efficiently transfer N-acetylgalactosamine or galactose to the H antigen, leading to reduced A or B antigen expression (Huang et al., 2019). This advance provides a crucial foundation for systematically uncovering previously elusive non-coding variants (Gueuning et al., 2023). Furthermore, minigene splicing assays serve as a valuable tool for functional validation of these variants, thereby clarifying the molecular mechanisms by which splice-site variations lead to aberrant transcript formation (Sanoguera-Miralles et al., 2024).

In this study, we employed an integrated approach combining serological phenotyping, third-generation sequencing, *in silico* splicing prediction, *in vitro* minigene assays, and protein structure analysis to investigate a novel *ABO* variant (c.239 + 6T>C) identified in four unrelated individuals presenting with an A_3_ phenotype. By establishing a comprehensive genotype-splicing-phenotype correlation, we aim to elucidate how this intronic variant disrupts normal mRNA splicing, resulting in reduced A antigen expression. This work will not only clarify the immunogenetic basis of this recurrent A_3_-associated variant but also provide a valuable framework for the precise molecular diagnosis and investigation of ABO blood group subtypes in clinical practice.

## Materials and methods

### Blood sample collection

Four unrelated, healthy blood donors exhibiting mixed-field agglutination were included in this study. None had a history of blood transfusion or familial genetic disorders. After providing informed consent, 2 mL of peripheral blood was collected from each participant into EDTA-coated tubes.

### ABO blood grouping

ABO blood grouping was performed using both the standard tube test and gel column agglutination. The standard tube test used anti-A, anti-B, anti-H, and anti-A_1_ reagents (Shanghai Hemo-pharmaceutical Biological Company) for forward typing, and A_1_, B, O, and A_2_ cells (Shanghai Hemo-pharmaceutical Biological Company) for plasma antibody identification in reverse typing. Gel column agglutination testing was performed with blood group gel cards (BIO-RAD, Switzerland) according to the manufacturer’s instructions, with reverse typing using A_1_ and B standard red blood cells (Shanghai Hemo-pharmaceutical Biological Company).

### DNA extraction and quantification

Genomic DNA was isolated from EDTA-anticoagulated peripheral blood using TIANGEN Blood DNA Kit (for whole blood samples, TIANGEN Biotech, China). DNA concentration and quality were measured by NanoDrop 2000/2000c spectrophotomer (Thermo Fisher Scientific, Waltham, MA, United States).

### Short tandem repeat (STR) analysis

Short tandem repeat (STR) genotyping was performed using the PowerPlex® 21 System (Promega Corporation, United States) according to the manufacturer’s instructions. A total of 20 autosomal STR loci together with the amelogenin (AMEL) locus were amplified using peripheral blood genomic DNA as the template. PCR products were separated by capillary electrophoresis on an ABI 3500 Genetic Analyzer (Applied Biosystems, United States), and allele calling was performed using GeneMapper™ ID-X software (Applied Biosystems, United States).

### Sanger sequencing

The PCR reaction was performed in a total volume of 40 μL containing 1× DNA polymerase mix (Qingke, Beijing, China) with 1 U of high-fidelity DNA polymerase, 200 μM of each dNTP, and 2.0 mM Mg^2+^, as well as 0.2 μM of each primer and approximately 100 ng of genomic DNA. The primer sequences for amplification and sequencing, as well as the PCR amplification parameters, were based on protocols described in a previous study (Zhu et al., 2010). The resulting sequences were analyzed using SeqMan software (Lasergene package, DNASTAR Inc., Madison, WI, United States). The ABO*A1.01 allele sequence was used as the reference. Differences from this reference sequence are denoted with arrows.

### Full-length ABO gene third-generation sequencing (three-fragment long-range PCR method)

**FIGURE 1:**
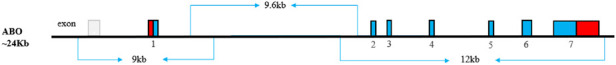
Schematic representation of the three-fragment amplification strategy for the *ABO* gene. The *ABO* gene spans approximately 24 kb. Blue rectangles represent the coding regions (exons 1–7), and red rectangles represent the 5′and 3′untranslated regions (UTRs). The three overlapping amplicons (9.0 kb, 9.6 kb, and 12.0 kb) are indicated by horizontal bars within the gene structure.

To cover the full-length ABO gene, including approximately 5 kb upstream of exon 1 and 3 kb downstream of exon 7, three pairs of overlapping primers (LR1, LR2, LR3) (Supplementary Table S1) were designed to generate amplicons of 9.0 kb, 9.6 kb, and 12.0 kb, respectively. Adjacent amplicons overlapped by approximately 1.3 kb, enabling the discrimination of the two complete haplotypes based on the SNPs present in the overlapping regions (Figure 1). The reaction mixture and cycling conditions are detailed in Supplementary Tables S2, S3, respectively.

1. Library Preparation and ONT Sequencing
  PCR amplicons were purified with AMPure XP beads (0.9:1 ratio). DNA end repair was performed using the NEBNext Ultra II End Repair/dA-Tailing module (30 µL), followed by 1:1 bead purification and barcode ligation (EXP-NBD114, Oxford Nanopore Technologies, ONT) with Blunt/TA Ligase Master Mix (NEB). Barcode-indexed libraries were purified (1:1 ratio), pooled according to concentrations measured by the Qubit dsDNA HS assay, and ligated with Adapter Mix (AMII, ONT) in Ligation Buffer (LNB) using NEBNext Quick T4 DNA Ligase. After final purification (0.4:1 ratio) and Long Fragment Buffer washes, the library was eluted into 15 µL of Elution Buffer. A median of 302 ng (range: 84–552 ng) of library was loaded onto R10.4 flow cells (FLO-PRO114M, ONT) and sequenced for 48 h using the SQK-NBD114-96 protocol. Sequencing was controlled by MinKNOW v24.02.16 on a 16-core Intel Xeon Silver 4314 CPU. Basecalling and demultiplexing were performed with Dorado v7.3.11 (super-accurate model at 400 bps) using a 24-thread, 64 GB RAM system with an NVIDIA RTX 4090 GPU.
2. Data Analysis and DNA Variant Calling
  Raw reads were demultiplexed, and barcodes were automatically identified. For ABO genotyping, reads were aligned to the T2T-CHM13v2.0 genome and corrected using DAFEI Biotechnology software. FASTQ reads for each ABO allele were extracted with bedtools (v2.30.0), and a custom Python workflow converted them into BAM file clusters. Reads were re-aligned to the ISBT ABO reference gene using minimap2. Variants, including SNVs and small indels, were identified with DeepVariant v1.2.0. Alignments and genotypes were visualized in the Integrative Genomics Viewer. Final haplotypes, including crossover regions, were generated by DAFEI Biotechnology (Guangzhou, China). Results were compared to ISBT ABO alleles v4.1 across different serological phenotypes.
3. Sequencing and variant analysis
  All four samples were successfully sequenced with a mean depth of 450× across the full-length ABO gene (range 210–680×). After applying the filtering criteria (depth ≥10, Phred quality ≥20, both strands supported), no additional pathogenic variants or structural rearrangements were detected apart from c.239 + 6T>C. Low-confidence calls that were excluded predominantly consisted of short insertions or deletions within homopolymeric tracts (≥4 identical nucleotides), a well-known systematic error mode of nanopore sequencing. These artifactual calls were manually inspected using IGV and removed when they showed strand bias or lacked clear signal transitions. No other unexpected or off-target variants were confirmed.

### Bioinformatic analysis

We employed the Rare Disease Data Center (RDDC) online RNA Splicer Tool (https://rddc.tsinghua-gd.org/zh), SpliceRover (http://bioit2.irc.ugent.be/rover/splicerover), and SpliceAPP (https://bc.imb.sinica.edu.tw/SpliceAPP/prediction.html) to predict the splicing patterns of potential splice variants. Furthermore, secondary structures of the wild-type (WT) and mutant (MT) glycosyltransferase proteins were generated using the online tool NovoPro (https://www.novopro.cn/tools/secondary-structure-prediction.html). Three-dimensional (3D) structural models were subsequently constructed using the Swiss-Model server (https://swissmodel.expasy.org/).

### Minigene functional validation

To determine the effect of the c.239 + 6T>C variant on ABO mRNA splicing, a minigene splicing reporter assay was performed. Briefly, a wild-type minigene construct spanning ABO exons 4-6 and containing flanking intronic sequences (including the splice donor site of intron 5) was generated. The variant was then introduced by site-directed mutagenesis, and the splicing patterns of the wild-type and mutant constructs were compared after transient transfection into HEK293T cells.

**FIGURE 2:**
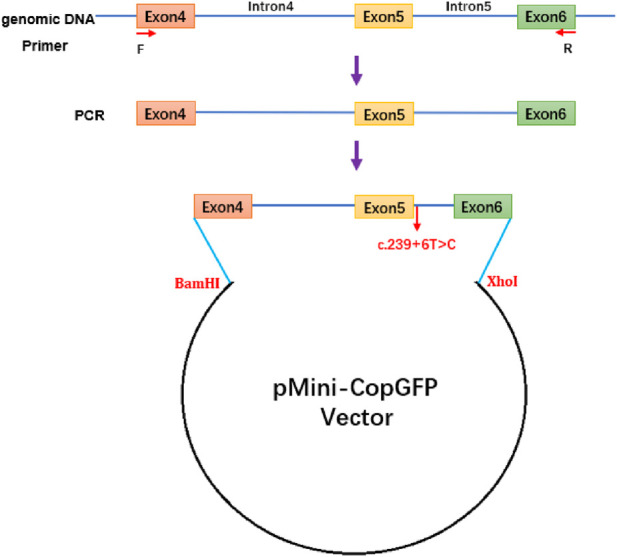
Flowchart for the Construction of c.239 + 6T>C Mutant Minigene Plasmid.

1. Construction of wild-type and mutant minigene plasmids
  Construction of wild-type minigene plasmid: A genomic segment spanning ABO exons 4-6 with flanking intronic sequences (∼4.25 kb) was amplified from a healthy control individual using primers that incorporated restriction sites. The primer, reaction mixture and cycling conditions are detailed in Supplementary Tables S4-S6, respectively.
  The PCR product was purified by 1.5% agarose gel electrophoresis followed by gel extraction using a Gel Extraction Kit (TIANGEN, DP209). The purified PCR product was double-digested with BamHI and XhoI and inserted into the pMini-CopGFP vector (Hitrobio) using a seamless cloning kit (Beyotime). Positive clones were confirmed by Sanger sequencing.
  Construction of mutant minigene plasmid: The c.239 + 6T>C mutation was introduced into the wild-type plasmid via site-directed mutagenesis PCR. The primer, reaction mixture and cycling conditions are detailed in Supplementary Tables S7-S9, respectively.
  The PCR product was purified by 1.5% agarose gel electrophoresis followed by gel extraction using a Gel Extraction Kit (TIANGEN, DP209). The purified linearized product was self-circularized using a seamless cloning kit (Beyotime) and transformed into *E. coli* Top10 competent cells. Positive clones were confirmed by Sanger sequencing. Figure 2 outlines the construction of the mutant minigene plasmid.
2. Cell transfection and RT-PCR
  HEK293T cells were seeded at 2–3 × 10^5^ cells per 35 mm dish. When cultures reached 50%–60% confluence on the following day, cells were transfected with 4 μg of wild-type or mutant minigene plasmid using Lipofectamine 2000 (Invitrogen) according to the manufacturer’s instructions. At 48 h post-transfection, total RNA was extracted with TRIzol reagent and reverse-transcribed into cDNA using the HiScript II First Strand cDNA Synthesis Kit (Vazyme). RT-PCR was then performed. The primer, reaction mixture and cycling conditions are detailed in Supplementary Tables S10-S12, respectively.
3. Analysis of RT-PCR products
  RT-PCR products were analyzed by two complementary methods. First, for routine analysis, products were resolved on a 1.5% agarose gel stained with GelRed at constant voltage for 30–40 min. Gel images were captured to assess amplification specificity, and bands of interest were excised, purified, and subjected to Sanger sequencing to confirm the identity of the splicing products at nucleotide resolution.

Second, to precisely determine transcript lengths and relative abundances, the same RT-PCR products were analyzed by capillary electrophoresis. The forward primer used in the RT-PCR reaction was labeled with 6-FAM at its 5’ end. In brief, an aliquot of the RT-PCR product was mixed with an appropriate volume of loading buffer, denatured at 95 °C for 5 min, and immediately cooled on ice. Capillary electrophoresis was then performed using a genetic analyzer, and transcript sizes were determined by comparison with an internal size standard. The relative abundance of each splicing variant was calculated as the ratio of its peak area to the total peak area of all detected transcripts.

## Results

### Serological results

**FIGURE 3:**
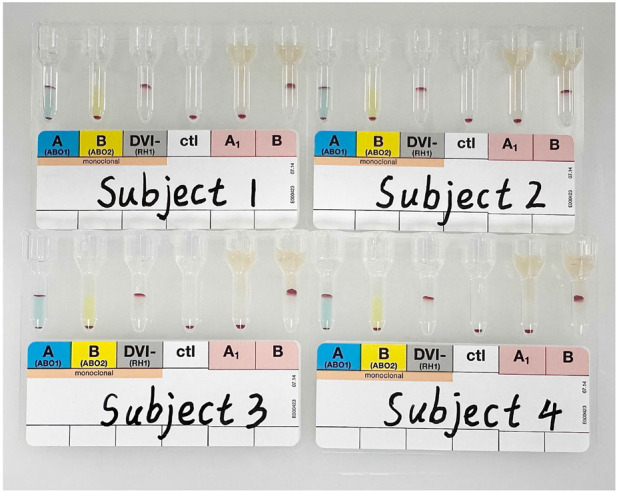
Serological phenotyping by gel column agglutination. All four subjects showed consistent results: forward typing showed anti-A double population (2+, ∼50% each) and anti-B negative; reverse typing showed strong agglutination with B cells (range 4 + ^w^ ∼ 4+) but not with A_1_ cells.

The ABO blood group serology results for the four subjects are presented in Table 1. By the tube method, forward typing revealed 2+ mixed-field agglutination with anti-A and no reaction with anti-B for all subjects; in reverse typing, all subjects showed 4+ agglutination with B cells and no agglutination with O or A_2_ cells. Reactivity with A_1_ cells, however, was variable: three subjects were non-reactive, while Subject 1 showed weak (1 + ^w^) agglutination. Additionally, all subjects were non-reactive with anti-A_1_ lectin but exhibited strong (4+) reactions with anti-H. Consistent with these findings, gel column agglutination also showed double population (2+, ∼50% each)) reactions with anti-A and no reaction with anti-B in forward typing. In reverse typing, gel column results similarly demonstrated 4+ agglutination with B cells (Subjects 1, 3, and 4 showed 4 + ^w^) and no agglutination with A_1_ cells or the control (Figure 3). Collectively, these serological patterns are characteristic of the A_3_ subgroup, with inconsistent anti-A_1_ production.

**TABLE 1:** Results of ABO serological grouping by tube test and gel column agglutination technology.

Sample	Forward typing (Tube method)				Forward typing (Gel column)		Reverse typing (Tube method)				Reverse typing (Gel column)		
	Anti-A	Anti-B	Anti-A1	Anti-H	Anti-A	Anti-B	A1c	A2c	Bc	Oc	A1c	Bc	Oc
Subject 1	2+^mf^	0	0	4+	2+^dcp^	0	1+^W^	0	4+	0	0	4+^W^	0
Subject 2	2+^mf^	0	0	4+	2+^dcp^	0	0	0	4+	0	0	4+	0
Subject 3	2+^mf^	0	0	4+	2+^dcp^	0	0	0	4+	0	0	4+^W^	0
Subject 4	2+^mf^	0	0	4+	2+^dcp^	0	0	0	4+	0	0	4+^W^	0

mf, mixed-field agglutination; dcp, double population (indicating mixed-field reaction in gel column); w, weak reaction.

### Sanger sequencing results

**FIGURE 4:**
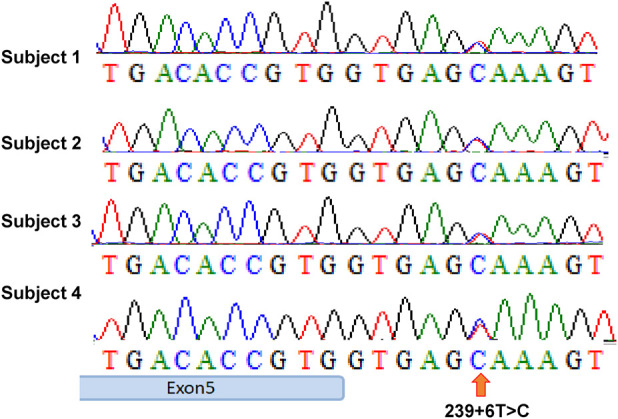
Sanger sequencing of *ABO* exons 5-7 in Subjects 1–4. All four subjects carried an identical c.239 + 6T>C variant.

Sanger sequencing was initially performed on exons 5-7 of the *ABO* gene in the four subjects. The results revealed no variants within exons 5, 6, or 7 in the context of an ABO*A1.01 allelic background. However, an identical c.239 + 6T>C variant within intron 5 was identified in all individuals (Figure 4).

### Single-molecule nanopore sequencing results

Nanopore sequencing revealed that, across all four samples, no variants were identified in the exonic or other intronic regions of the ABO*A1.01 haplotype, with the sole exception of a single-base substitution located in the intronic region adjacent to the exon 5 splice site (c.239 + 6T>C). As summarized in Table 2, a novel ABO*A1.01-like allele was identified in all four subjects, defined by the c.239 + 6T>C variant. The other allele was ABO*O.01.01 in Subjects 1 and 2, and ABO*O.01.02 in Subjects 3 and 4.

**TABLE 2:** Results of nanopore single-molecule sequencing of the ABO gene.

Sample	Gene	Haplotype 1			Haplotype 2			Phenotype
		Pheno-type	Allele	Mutation	Pheno-type	Allele	Mutation	
Subject 1	ABO	0	ABO*O.01.01	c.261delG;	A3	ABO*A.NEW	c.239+6T>C	A3
Subject 2	ABO	0	ABO*O.01.01	c.261delG;	A3	ABO*A.NEW	c.239+6T>C	A3
Subject 3	ABO	0	ABO*O.01.02	c.106G>T;c.188G>A; c.189C>T; c.220C>T; c.261delG;c.297A>G; c.646T>A;c.681G>A; c.771C>T; c.829G>A	A3	ABO*A.NEW	c.239+6T>C	A3
Subject 4	ABO	0	ABO*O.01.02	c.106G>T; c.188G>A; c.189C>T; c.220C>T; c.261delG; c.297A>G; c.646T>A; c.681G>A; c.771C>T;c.829G>A	A3	ABO*A.NEW	c.239+6T>C	A3

### Short tandem repeat analysis results

**FIGURE 5:**
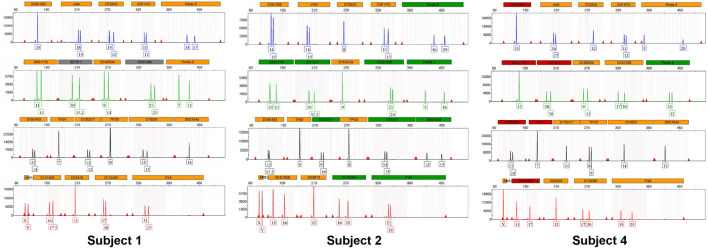
STR profiles of Subject 1, Subject 2, and Subject 4. All three subjects showed one or two allelic peaks at each of the 20 autosomal loci, with no locus displaying three or more alleles.

Due to sample depletion, STR analysis could not be performed for Subject 3. For the remaining three subjects (Subjects 1, 2, and 4), STR analysis was performed to exclude blood group chimerism as a cause of the mixed-field agglutination. All three STR profiles showed exclusively single (homozygous) or two peaks (heterozygous) at each of the 20 autosomal loci examined, with no locus exhibiting three or more peaks and no significant peak height or area imbalance across the profile (Figure 5)-findings consistent with the absence of chimerism.

### *In silico* splicing analysis

**FIGURE 6:**
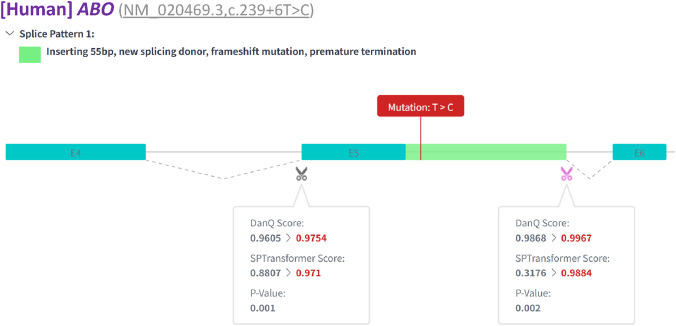
Splicing analysis using the RDDC RNA Splicer tool for the *ABO* c.239 + 6T>C variant: The variant generates a cryptic donor site 55 bp downstream of the intron 5 donor site, leading to a 55 bp insertion, frameshift mutation, and premature termination. DanQ and SPTransformer scores range from 0 to 1, with values closer to 1 indicating a higher likelihood of being a true splice site.

Splicing analysis using the RDDC RNA Splicer tool revealed that the novel ABO variant (c.239 + 6T>C) generates a cryptic donor site located 55 nucleotides downstream of the canonical 5′splice site of intron 5, which is predicted to result in aberrant splicing. Splice-altering potential scores (DanQ and SPTransformer) increased significantly following the variant: in intron 5, DanQ rose from 0.9868 to 0.9967, and SPTransformer from 0.3176 to 0.9884 (Figure 6).

These findings were further supported by SpliceRover and SpliceAPP. SpliceRover scored the wild-type (WT) at 0.570276 and the mutant (MT) at 0.953626, suggesting activation of this cryptic donor site (Table 3). SpliceAPP also predicted a significant splicing alteration for this variant, with a score of 0.1903, which exceeds the official 5′ss model threshold of 0.13, indicating a high likelihood of disrupted canonical splicing (Table 4). Collectively, these *in silico* predictions are highly consistent and indicate that c.239 + 6T>C creates a cryptic donor site 55 nt downstream of the canonical 5′splice site of intron 5.

**TABLE 3:** Prediction of wild-type (WT) and mutant (MT) splicing probability by SpliceRover.

SpliceRover	Hit (with context)	Score	Prediction
WT	TGAAG|GTATTAG	0.570276	Baseline
MT	TGAAG|GTATTAG	0.953626	Cryptic donor activated

Scores range from 0 to 1; values closer to 1 indicate a higher likelihood of being a true splice site; “|”: a cryptic donor site located 55 nucleotides downstream of the 5′ splice site of intron 5.

**TABLE 4:** SpliceAPP prediction of splicing effects for the c.239+6T>C variant.

Prediction tool	Variant	Model	Distance to 5'ss	Score	Effect
SpliceAPP	ABO:c.239 + 6T>C	5' ss	6 nt	0.1903	Significant

Scores range from 0 to 1; Score > 0.13 (official threshold for the 5‘ ss model) indicates a significant splicing alteration.

### Minigene splicing assay

**FIGURE 7:**
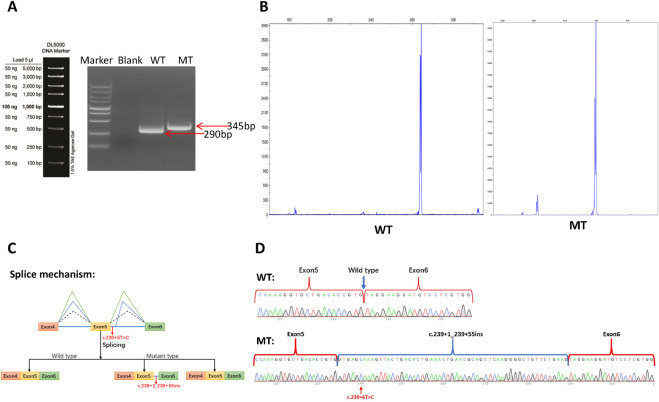
Validation of aberrant splicing for the *ABO* c.239+6T>C variant by minigene splicing assays. **(A)** RT-PCR gel electrophoresis results: WT shows a 290 bp band; MT shows a 345 bp band, indicating a 55 bp insertion. **(B)** Capillary electrophoresis results: WT shows a 288.65 bp fragment; MT shows a 344.62 bp fragment along with a small amount of the 288.65 bp normal transcript. A small amount of aberrant transcripts was detected in the wild-type sample, which is consistent with the expected background level. The x-axis shows product length (bp); the y-axis shows relative fluorescence units. **(C)** Splicing pattern of the c.239 + 6T>C variant. Aberrant pattern: c.239 6T>C disrupts the canonical donor splice site of intron 5, activating a cryptic donor site 55 bp downstream, resulting in a 55 bp intronic insertion between exons 5 and 6. Normal pattern: Normal splicing. **(D)** Sequencing results: WT shows the normal exon 5–6 junction; MT shows a 55 bp intronic retention (c.239 + 1_239 + 55).

We performed an *in vitro* minigene assay to definitively characterize the impact of the *ABO* c.239 + 6T>C variant on RNA splicing. RT-PCR gel electrophoresis results revealed a single 290 bp (vector: 71 bp, exons 4–6: 48 + 36+135 bp; total 290 bp) product from the wild-type (WT) construct, whereas the mutant (MT) produced a longer 345 bp band (Figure 7A). Capillary electrophoresis further resolved these products with higher precision: the WT construct yielded a normal transcript of 288.65 bp; the MT construct yielded a predominant aberrant transcript of 344.62 bp along with a minor normal transcript of 288.65 bp, indicating that the variant severely impairs but does not completely abolish canonical splicing (Figure 7B). Sanger sequencing confirmed normal splicing in the WT, while in the MT, it revealed that the aberrant product resulted from the retention of a 55-nucleotide intronic fragment (Figure 7D). Due to the extremely low abundance of the normal transcript in the MT sample, Sanger sequencing failed to detect it. Mechanistic analysis demonstrated that the c.239 + 6T>C variant generates two splicing patterns. The aberrant splicing pattern disrupts the canonical donor splice site at the 5′end of intron 5, activating a cryptic donor site 55 bp downstream and resulting in a 55 bp intronic insertion between exons 5 and 6, whereas the normal splicing pattern produces the correctly spliced transcript (Figure 7C). The aberrant 55 bp intronic insertion shifts the reading frame and introduces a premature termination codon (p.Cys80TrpfsTer2). Amino acid alignment reveals the resultant frameshift and early termination (Supplementary Figure S1A). Furthermore, structural modeling using the Swiss-Model server demonstrates that, in contrast to the fully folded wild-type (WT) enzyme, the mutant (MT) protein is drastically truncated and lacks essential functional domains (Supplementary Figure S1B).

## Discussion

Accurate ABO blood typing is essential for transfusion compatibility, yet ABO subtypes often complicate identification. With the advent of third-generation sequencing technologies, splice-site variations have emerged as a key focus in ABO subtype research. Notably, these non-coding variants involve more complex mechanisms than coding variants, making their elucidation critical to understanding ABO subtype genesis (Yang et al., 2026; Hong et al., 2021). mRNA splicing is a critical step in eukaryotic gene expression. The spliceosome precisely excises non-coding regions and ligates coding regions to generate mature mRNA. This process depends on the recognition of consensus sequences, including the 5′splice site, 3′splice site, and branch point (Zhang et al., 2022). Variants that disrupt these sequences impair spliceosome recruitment, triggering aberrant splicing events such as exon skipping or intron retention (Abramowicz and Gos, 2019). Ultimately, these splicing defects compromise the production of authentic transcripts, impair glycosyltransferase synthesis, and disrupt antigen expression.

In this study, we identified four unrelated probands exhibiting mixed-field agglutination with anti-A, a hallmark of the A_3_ phenotype. Sanger sequencing and nanopore sequencing revealed that all four samples carried an ABO*A1.01 allele harboring a novel c.239 + 6T>C variant. The conserved GT-AG dinucleotide motif at splice sites is essential for accurate mRNA splicing (Lee and Rio, 2015). Although the c.239 + 6T>C variant does not alter the GT dinucleotide at the 5′splice site, previous studies have shown that substitutions at positions +5 and +6 can impair U1 snRNP binding to pre-mRNA, leading to aberrant splicing (Schmid et al., 2013). In two classic studies, Chen et al. dissected the c.155 + 5G>A (IVS3+5G>A) variant in a B_3_ individual and the c.374 + 5G>A (IVS6+5G>A) variant in an A_el_ individual, demonstrating that these variants drive aberrant splicing, predominantly generating transcripts with skipping of exons 3 and 6, respectively (Chen et al., 2015; Chen et al., 2012). Subsequently, Hong et al. identified a novel allele defining the B_el_ phenotype, harboring the c.28 + 5G>A variant (Hong et al., 2021). Shao et al. further demonstrated that the same variant, when placed in an ABO*A2.01 background, shifts the expected A_2_ phenotype to A_el_ (Shao et al., 2024). Both studies point to a common splicing defect characterized by partial intron 1 retention (Hong et al., 2021; Shao et al., 2024). Collectively, these studies establish that aberrant RNA splicing drives the genesis of these ABO subtypes.

Unlike the well-characterized splice-site variants in A_el_, B_el_, and B_3_ subtypes, those associated with the A_3_ phenotype have been rarely investigated. The novel c.239 + 6T>C variant represents the second splice-site variant linked to the A_3_ phenotype, following the c.98 + 3A>G variant previously reported by Yang et al. (Cai et al., 2013). To further characterize the c.239 + 6T>C variant, we conducted *in silico* splicing analyses using RDDC, SpliceRover, and SpliceAPP. All three tools predicted aberrant splicing. Critically, both RDDC (DanQ: 0.9967; SPTransformer: 0.9884) and SpliceRover (0.953626) indicated activation of a cryptic splice site 55 bp downstream of the canonical site, predicting the generation of an aberrantly elongated transcript retaining an intronic fragment. SpliceAPP yielded a score of 0.1903 (5’ss model, threshold >0.13), further supporting this prediction. Analysis of minigene splicing products by gel electrophoresis confirmed this prediction. The wild-type A allele transcript yielded a 290-bp band, whereas the c.239 + 6T>C variant produced a 345-bp band, consistent with the retention of a 55-bp intronic sequence. This aberrant splicing event introduces a premature stop codon, which is predicted to truncate the polypeptide to 80 amino acids. Similarly, the c.98 + 3A>G variant is predicted to result in exon skipping or intron retention, generating truncated polypeptides of 33 and 32 amino acids, respectively (Supplementary Figure S2). Importantly, transcripts bearing premature termination codons are typically recognized and degraded by the nonsense-mediated mRNA decay (NMD) pathway-a conserved eukaryotic mRNA surveillance mechanism (Behera et al., 2025). Consequently, these aberrant transcripts are unlikely to be translated. Even if a small fraction escape NMD, the resulting truncated GTA polypeptides lack the catalytic domain and are therefore predicted to be catalytically inactive.

However, the mixed-field agglutination observed in A_3_ red blood cells following incubation with anti-A reagent suggests reduced, rather than absent, A antigen expression on the cell surface. To investigate this discrepancy, we performed capillary electrophoresis on the minigene-derived RT-PCR products, a more sensitive method than agarose gel electrophoresis. Strikingly, this analysis uncovered a preponderance of aberrant transcripts with a 55 bp intronic retention, together with a scant population of correctly spliced normal transcripts. These correctly spliced normal transcripts are likely generated by residual activity of the canonical splice site and are expected to be translated into trace amounts of functional GTA (Bueno-Martínez et al., 2022). This minute quantity of GTA transfers A antigens onto a subset of red blood cells, thereby contributing to the A_3_ phenotype (Ogasawara et al., 2024; Liang et al., 2015). A similar mechanism has been described for the ABO*A2.01 allele carrying the c.28 + 5G>A variant. Minigene assay revealed that this variant generates not only an aberrant transcript (introducing a premature termination codon and yielding a nonfunctional GTA) but also a trace amount of a wild-type transcript that produces a functional GTA, thereby shifting the expected A_2_ phenotype to the weaker Ael phenotype (Shao et al., 2024). Collectively, these findings establish a refined mechanistic model for A_3_ phenotype generation: a splice-site variant that disrupts canonical splicing while permitting residual correct splicing, producing trace amounts of functional glycosyltransferase A sufficient for weak but detectable A antigen expression. This splicing paradigm may extend to other ABO subtypes and beyond.

Notably, anti-A_1_ antibodies were detected in one of the four A_3_ probands, possibly reflecting variations in immune response among individuals with the A_3_ phenotype (Daniels and Daniels, 2013). Regarding transfusion management, as the four subjects were blood donors, it was not possible to monitor the outcome of any transfusion. However, should future transfusion be required, it is advisable for these individuals to receive washed group O red blood cells, particularly for those who have developed anti-A_1_ antibodies, as transfusion of group A red blood cells should be avoided in these cases to minimize the risk of hemolytic transfusion reactions.

One limitation of this study is that, due to the unavailability of fresh blood samples, we were unable to directly examine the transcript status *in vivo* or validate GTA expression at the protein level.

## Conclusion

In conclusion, we identified a novel ABO*A1.01 (c.239 + 6T>C) allele responsible for the A_3_ phenotype in four unrelated Chinese blood donors. The nucleotide sequence of this allele has been submitted to GenBank under accession no. PV862049. By combining Sanger and nanopore sequencing with minigene splicing assays, we demonstrated that the c.239 + 6T>C variant causes both aberrant splicing and residual correct splicing. The former produces a truncated, inactive GTA, while the latter generates trace amounts of functional GTA. These two mechanisms together explain the A_3_ phenotype. Together, these findings expand the variant spectrum of the *ABO* gene and highlight the diagnostic value of integrating sequencing-based and functional assays for deciphering blood group subtypes. This study contributes to a deeper understanding of the immunogenetic basis of ABO variation, with implications for transfusion safety and personalized immunohematology.

## Data Availability

The data presented in this study have been deposited in the GenBank database (accession number PV862049; available at: https://www.ncbi.nlm.nih.gov/nuccore/PV862049).

## Supplementary material

SUPPLEMENTARY FIGURE 1 Structural analysis of wild-type and mutant GTA for the c.239 + 6T>C variant. **(A)** Secondary structures and amino acid sequences of wild-type and mutant. Helical regions are highlighted in red, and β-strand regions in blue. **(B)** Three-dimensional structural models of wild-type A glycosyltransferase and its truncated mutant.

SUPPLEMENTARY FIGURE 2 Prediction of aberrant splicing for the ABO c.98+3A>G variant by in silico prediction and structural analysis of mutant GTA. **(A)** The c.98+3A>G variant exhibits two distinct splicing patterns. Pattern 1: Activation of a cryptic splice donor site within intron 2, resulting in a 152 bp insertion and a consequent frameshift with premature termination. Pattern 2: Skipping of exon 2, also resulting in a frameshift and premature termination. **(B)** Secondary structures and amino acid sequences of GTA resulting from the two aberrant splicing patterns. Secondary structure predictions were generated using the online tool (https://www.novopro.cn/tools/secondary-structure-prediction.html).
