# Supplementary material for: A novel ABO splice site variant underlying the A3 phenotype: immunogenetic basis and functional dissection
Source: Front Genet. 2026 Jun 19;17:1839848. doi: 10.3389/fgene.2026.1839848 (PMC13327653; doi:10.3389/fgene.2026.1839848)
Supplement: Supplementary file 1 [file Presentation11.ppt]

## Slide 1
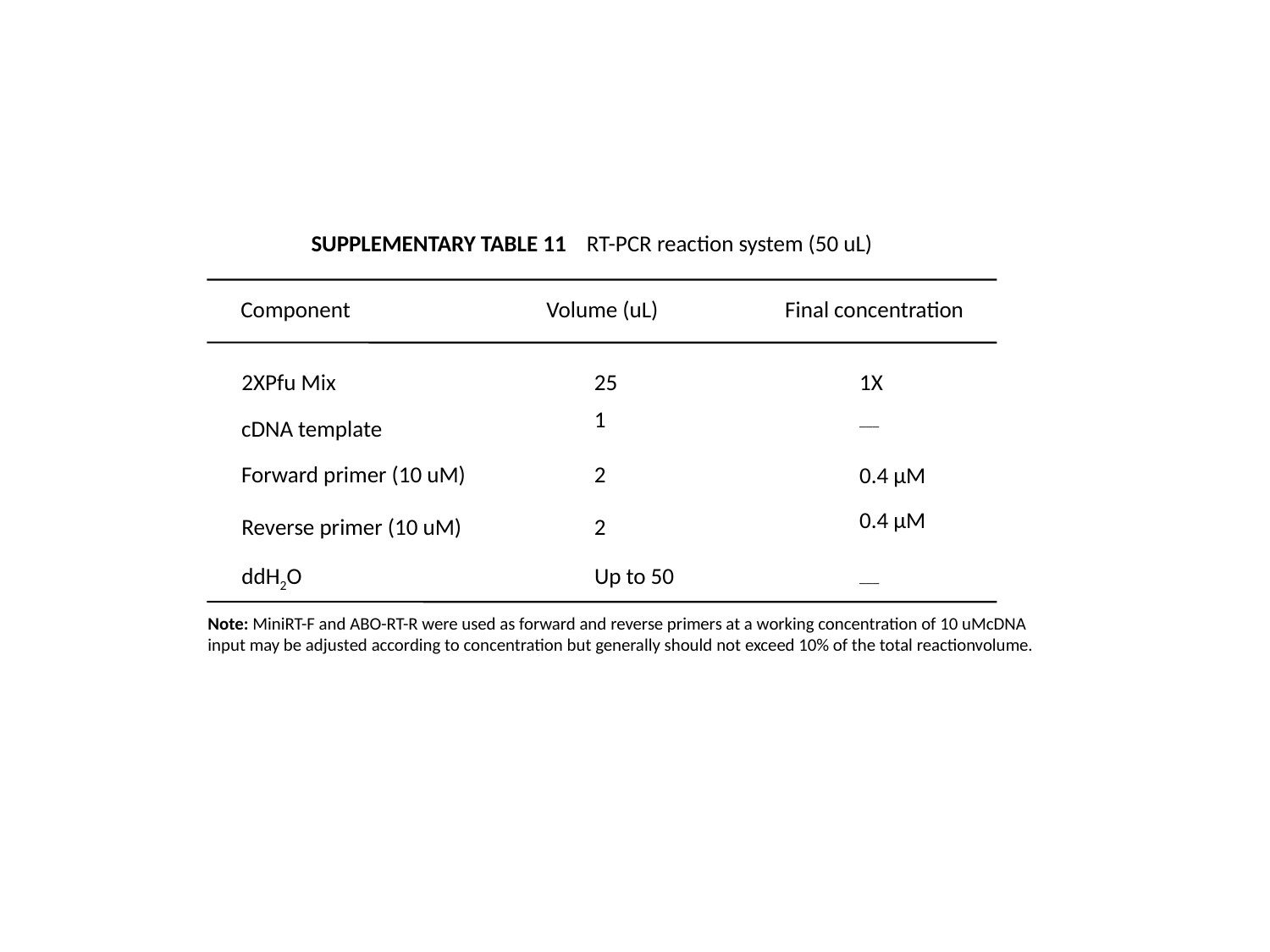

SUPPLEMENTARY TABLE 11 RT-PCR reaction system (50 uL)
Component
Volume (uL)
Final concentration
25
2XPfu Mix
1X
1
___
cDNA template
Forward primer (10 uM)
2
0.4 μM
0.4 μM
Reverse primer (10 uM)
2
ddH2O
Up to 50
___
Note: MiniRT-F and ABO-RT-R were used as forward and reverse primers at a working concentration of 10 uMcDNA input may be adjusted according to concentration but generally should not exceed 10% of the total reactionvolume.
