# Supplementary material for: A novel ABO splice site variant underlying the A3 phenotype: immunogenetic basis and functional dissection
Source: Front Genet. 2026 Jun 19;17:1839848. doi: 10.3389/fgene.2026.1839848 (PMC13327653; doi:10.3389/fgene.2026.1839848)
Supplement: Supplementary file 2 [file Presentation12.ppt]

## Slide 1
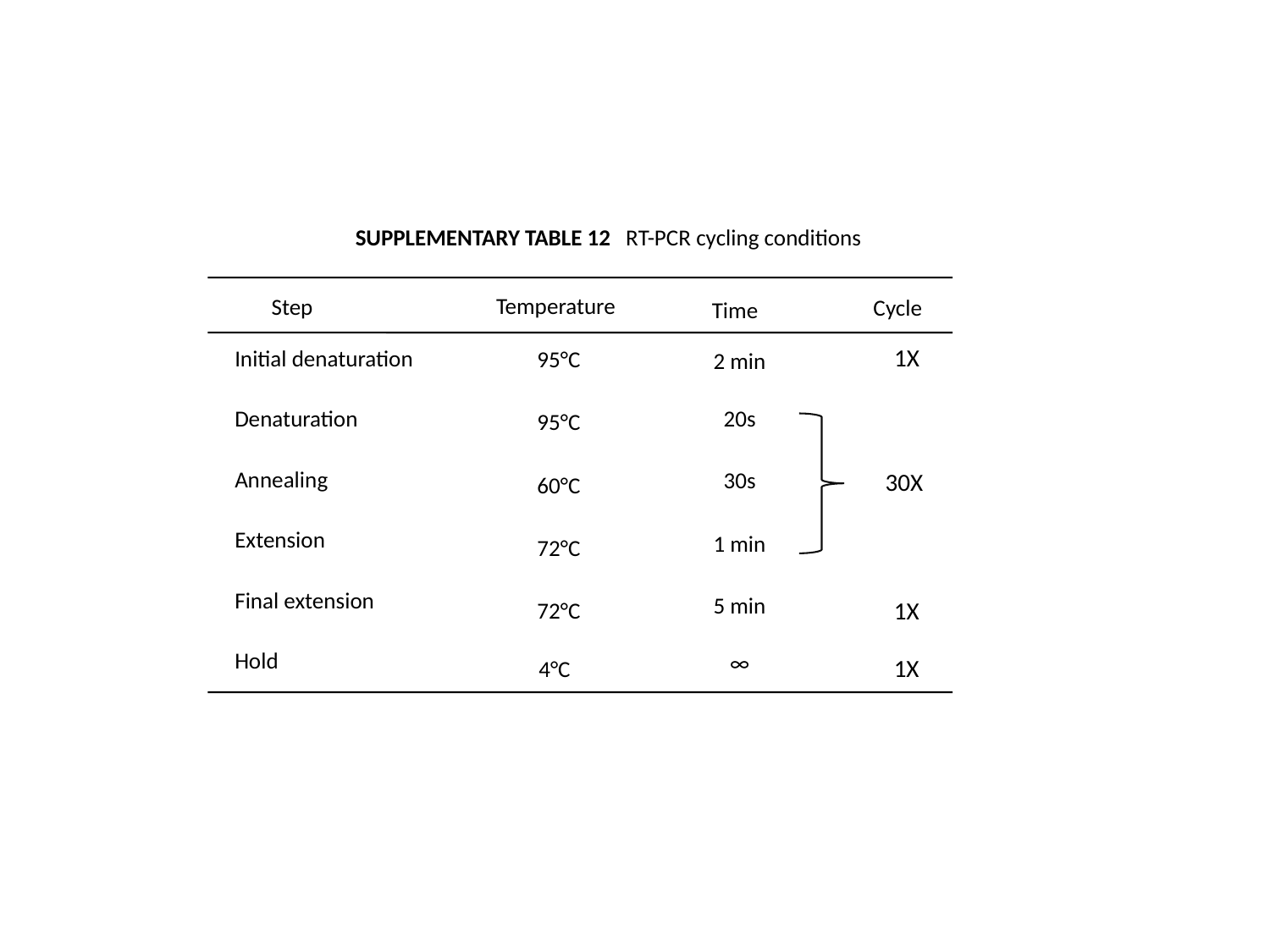

SUPPLEMENTARY TABLE 12 RT-PCR cycling conditions
Temperature
Step
Cycle
Time
1X
Initial denaturation
95°C
2 min
20s
Denaturation
95°C
30s
Annealing
30X
60°C
1 min
Extension
72°C
Final extension
5 min
1X
72°C
Hold
∞
1X
4°C
