# Supplementary material for: A novel ABO splice site variant underlying the A3 phenotype: immunogenetic basis and functional dissection
Source: Front Genet. 2026 Jun 19;17:1839848. doi: 10.3389/fgene.2026.1839848 (PMC13327653; doi:10.3389/fgene.2026.1839848)
Supplement: Supplementary file 4 [file Presentation2.ppt]

## Slide 1
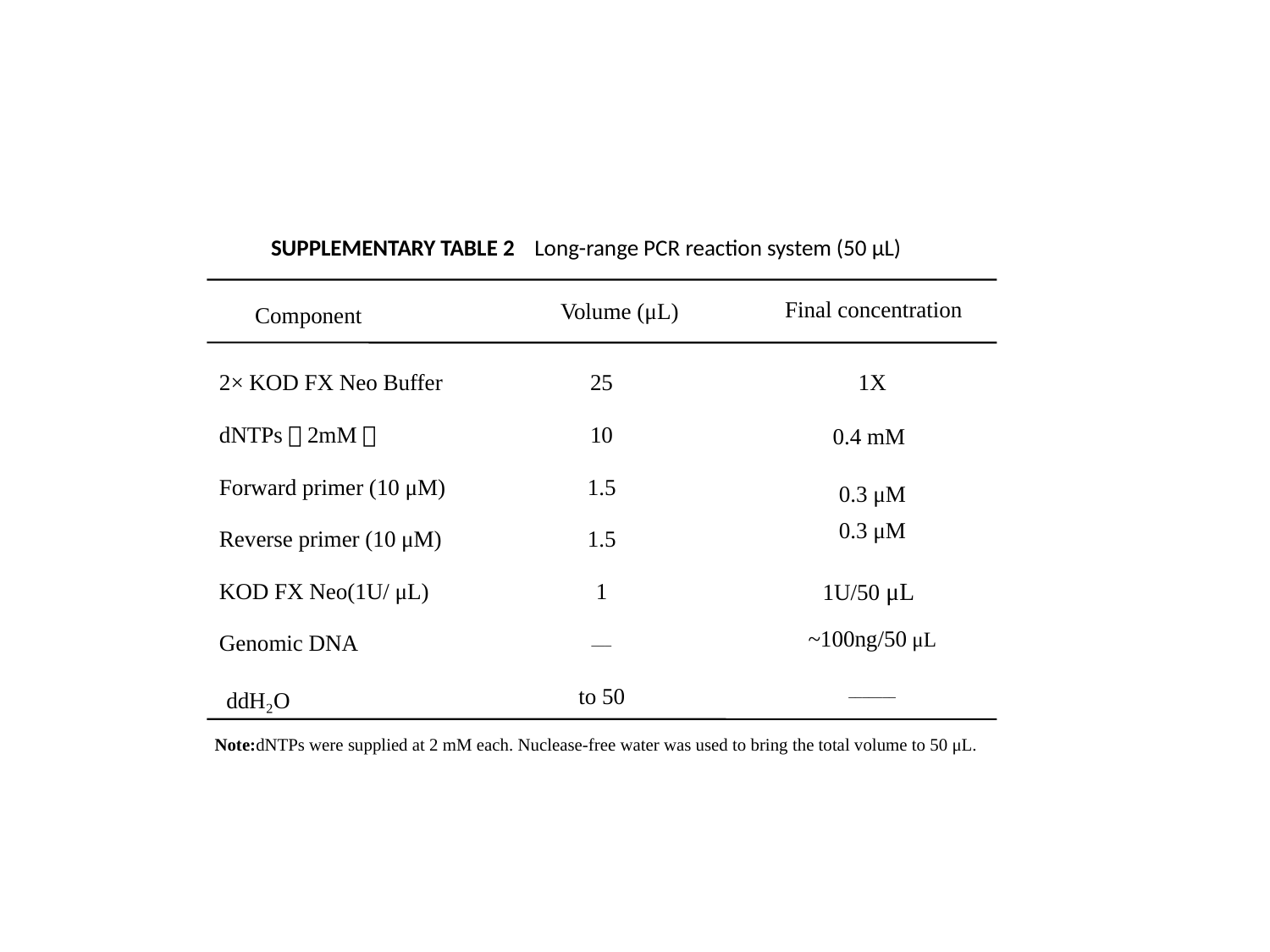

SUPPLEMENTARY TABLE 2 Long-range PCR reaction system (50 μL)
Final concentration
Volume (μL)
Component
25
2× KOD FX Neo Buffer
1X
0.4 mM
10
dNTPs（2mM）
Forward primer (10 μM)
1.5
0.3 μM
0.3 μM
Reverse primer (10 μM)
1.5
1U/50 μL
KOD FX Neo(1U/ μL)
1
~100ng/50 μL
Genomic DNA
___
_______
to 50
ddH₂O
Note:dNTPs were supplied at 2 mM each. Nuclease-free water was used to bring the total volume to 50 μL.
