# Supplementary figures and images for: A novel ABO splice site variant underlying the A3 phenotype: immunogenetic basis and functional dissection
Source: Front Genet. 2026 Jun 19;17:1839848. doi: 10.3389/fgene.2026.1839848 (PMC13327653; doi:10.3389/fgene.2026.1839848)

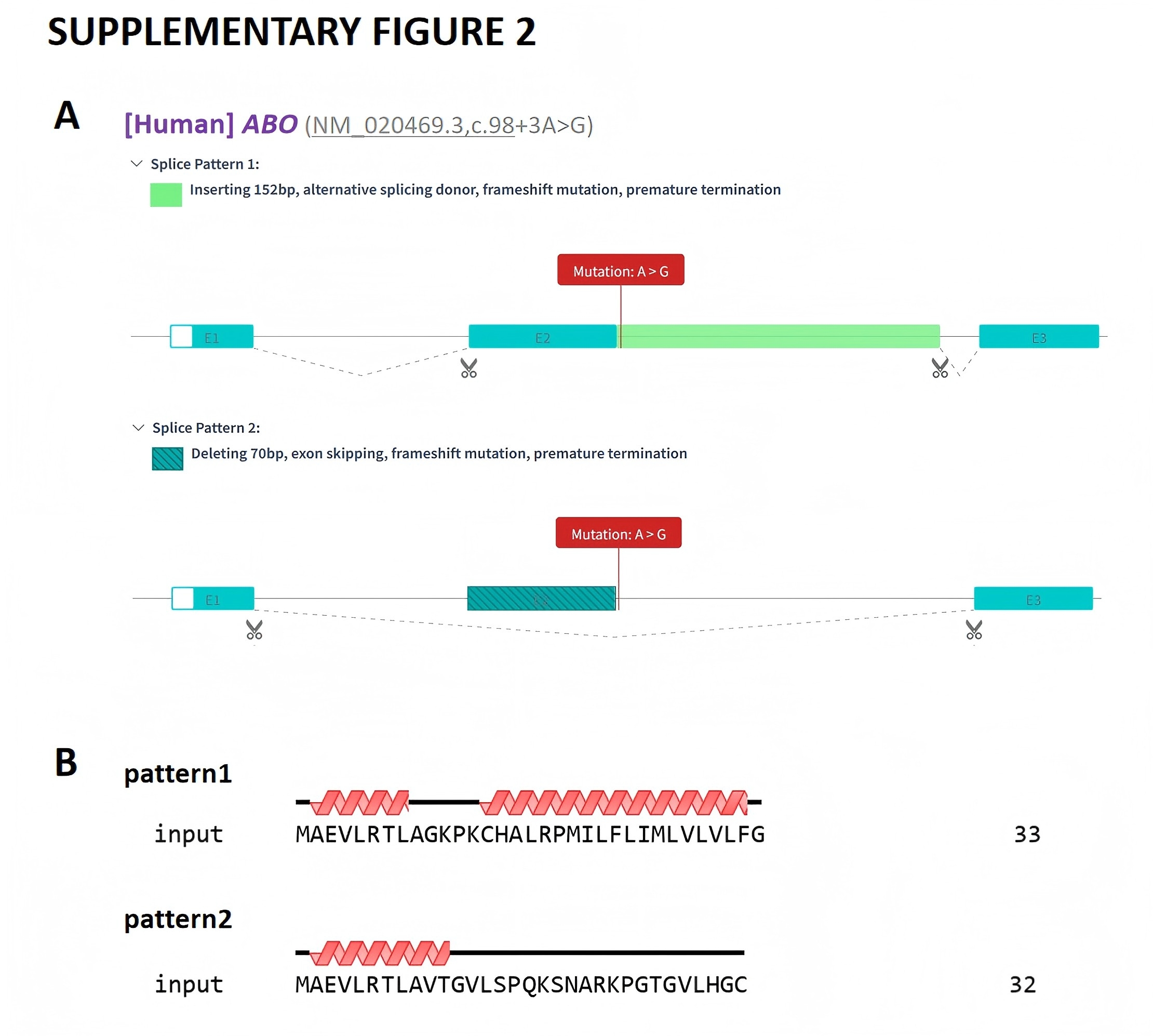

Supplement: Supplementary file 5 [file Image2.jpg]

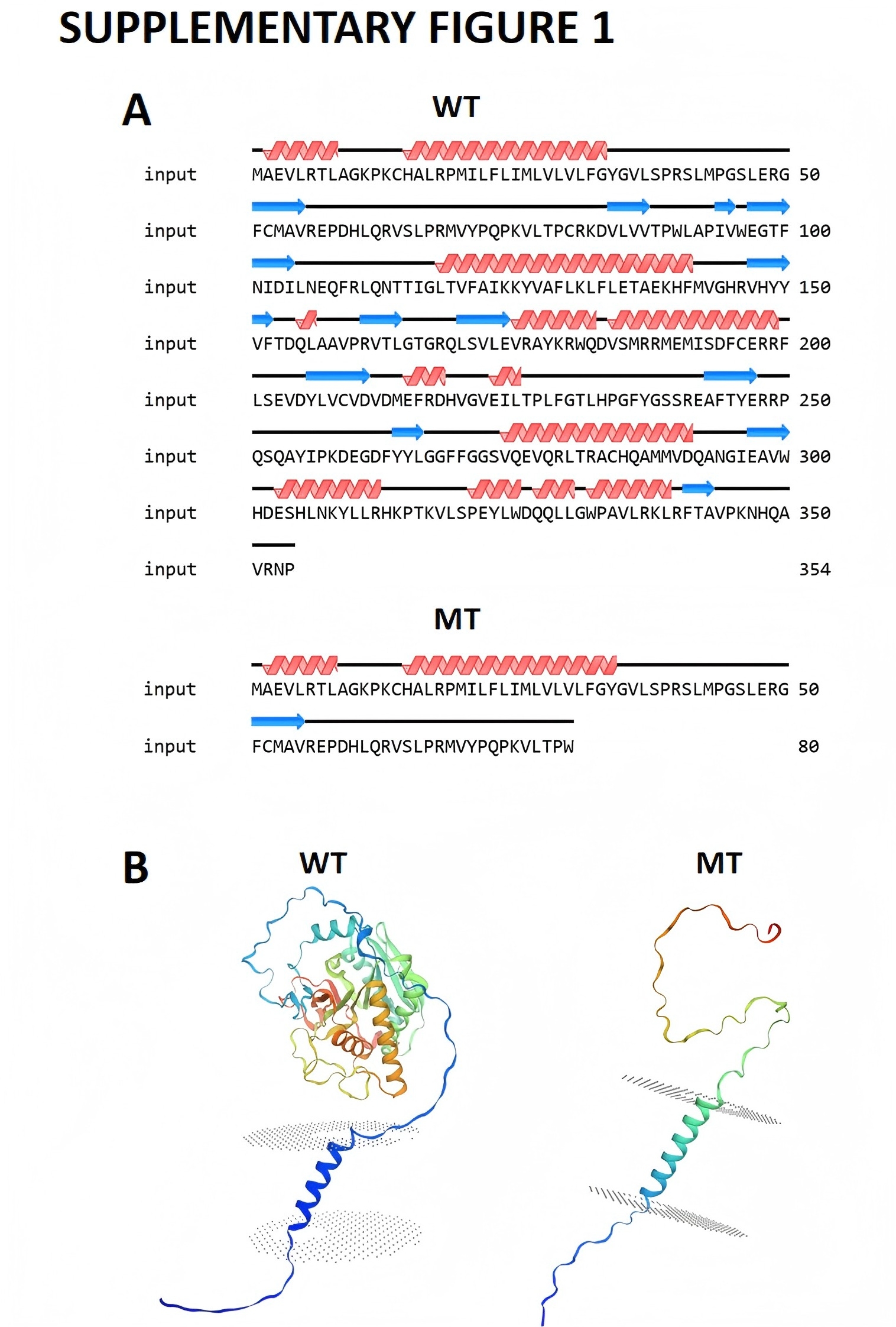

Supplement: Supplementary file 14 [file Image1.jpg]
