# Supplementary material for: A novel ABO splice site variant underlying the A3 phenotype: immunogenetic basis and functional dissection
Source: Front Genet. 2026 Jun 19;17:1839848. doi: 10.3389/fgene.2026.1839848 (PMC13327653; doi:10.3389/fgene.2026.1839848)
Supplement: Supplementary file 6 [file Presentation1.ppt]

## Slide 1
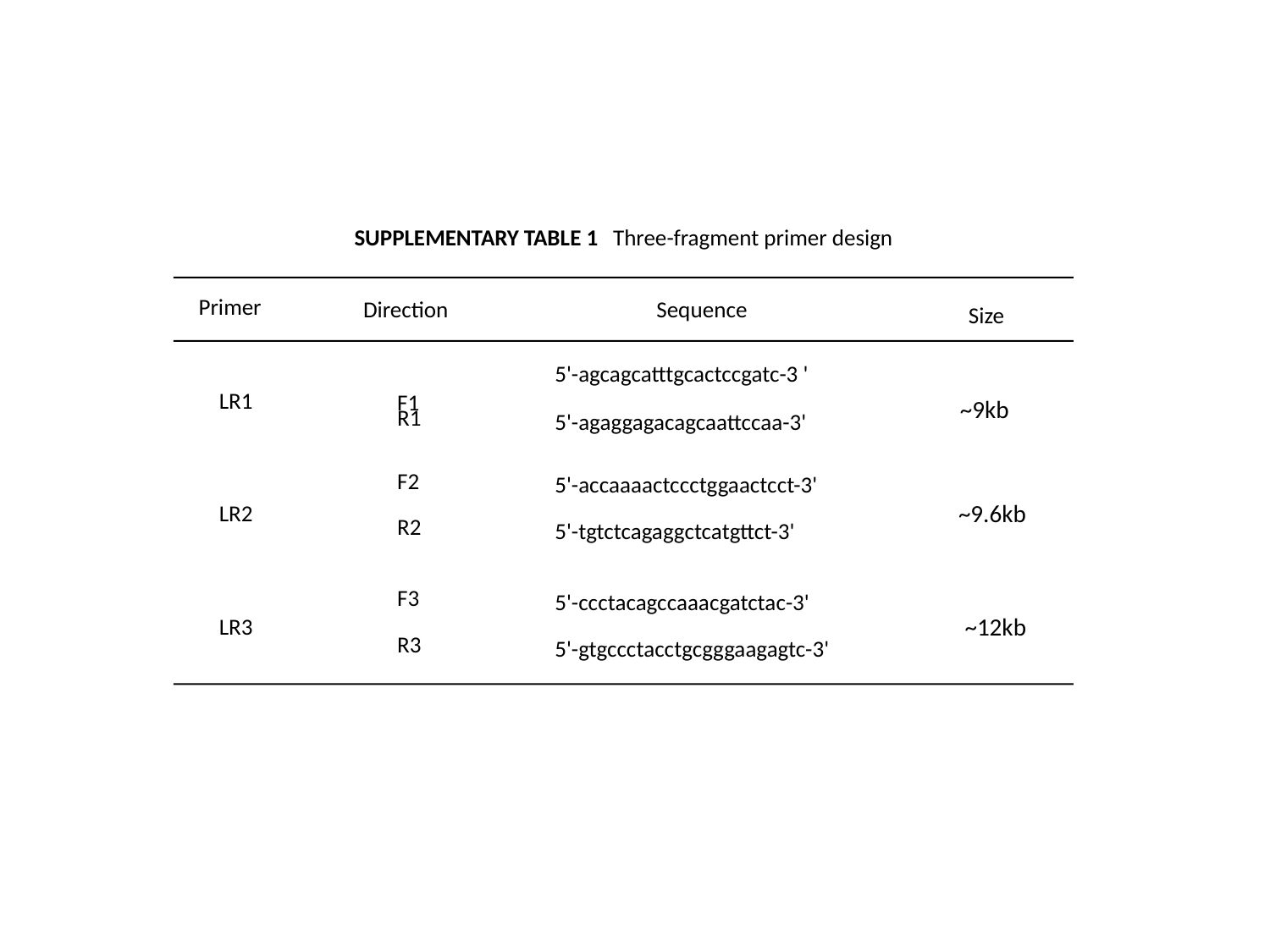

SUPPLEMENTARY TABLE 1 Three-fragment primer design
Primer
Sequence
Direction
Size
 F1
5'-agcagcatttgcactccgatc-3 '
LR1
~9kb
R1
5'-agaggagacagcaattccaa-3'
F2
5'-accaaaactccctggaactcct-3'
~9.6kb
LR2
R2
5'-tgtctcagaggctcatgttct-3'
F3
5'-ccctacagccaaacgatctac-3'
 ~12kb
LR3
R3
5'-gtgccctacctgcgggaagagtc-3'
