# Supplementary material for: A novel ABO splice site variant underlying the A3 phenotype: immunogenetic basis and functional dissection
Source: Front Genet. 2026 Jun 19;17:1839848. doi: 10.3389/fgene.2026.1839848 (PMC13327653; doi:10.3389/fgene.2026.1839848)
Supplement: Supplementary file 7 [file Presentation10.ppt]

## Slide 1
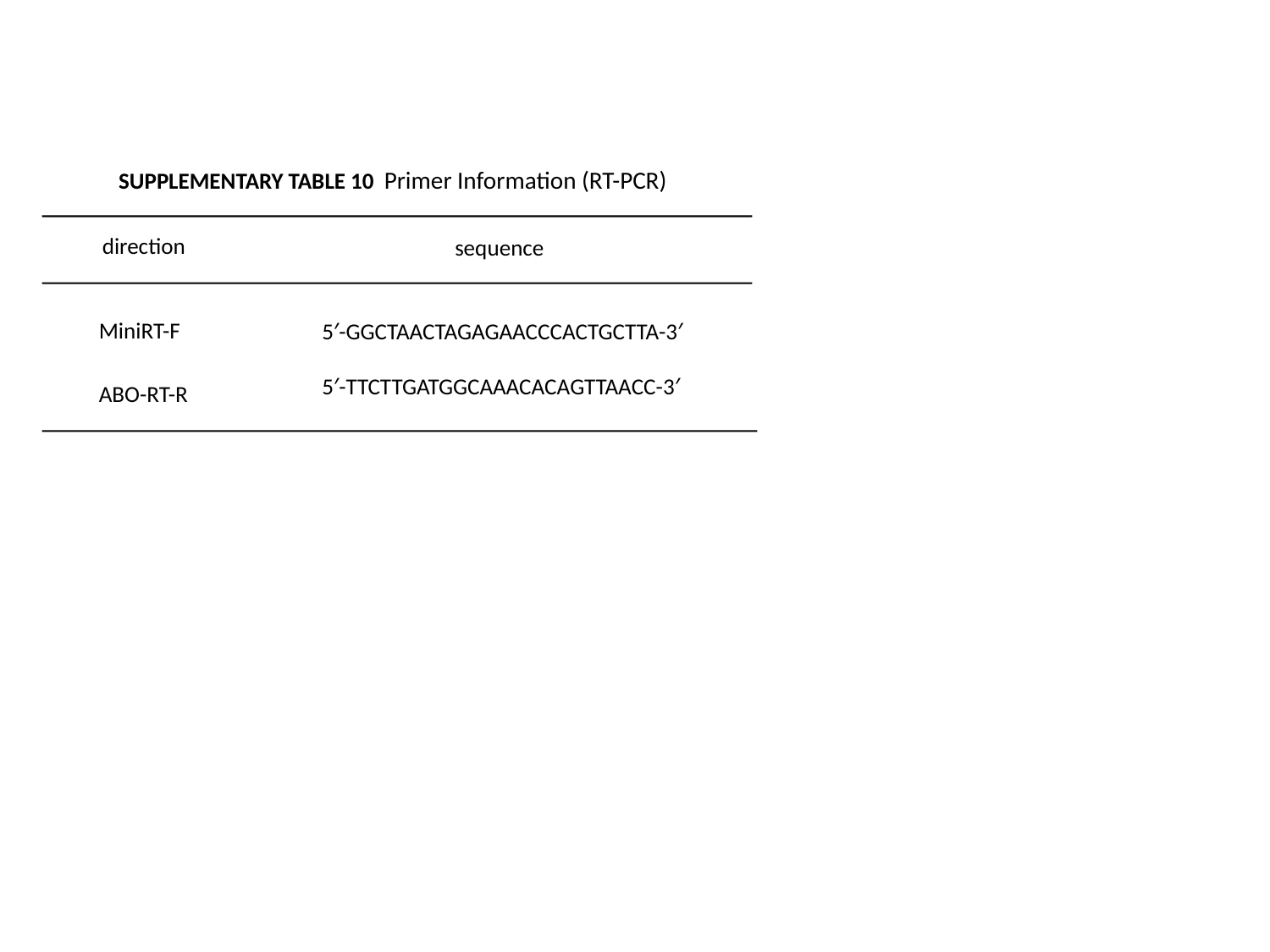

SUPPLEMENTARY TABLE 10 Primer Information (RT-PCR)
direction
sequence
5′-GGCTAACTAGAGAACCCACTGCTTA-3′
MiniRT-F
5′-TTCTTGATGGCAAACACAGTTAACC-3′
ABO-RT-R
