# Supplementary material for: A novel ABO splice site variant underlying the A3 phenotype: immunogenetic basis and functional dissection
Source: Front Genet. 2026 Jun 19;17:1839848. doi: 10.3389/fgene.2026.1839848 (PMC13327653; doi:10.3389/fgene.2026.1839848)
Supplement: Supplementary file 9 [file Presentation4.ppt]

## Slide 1
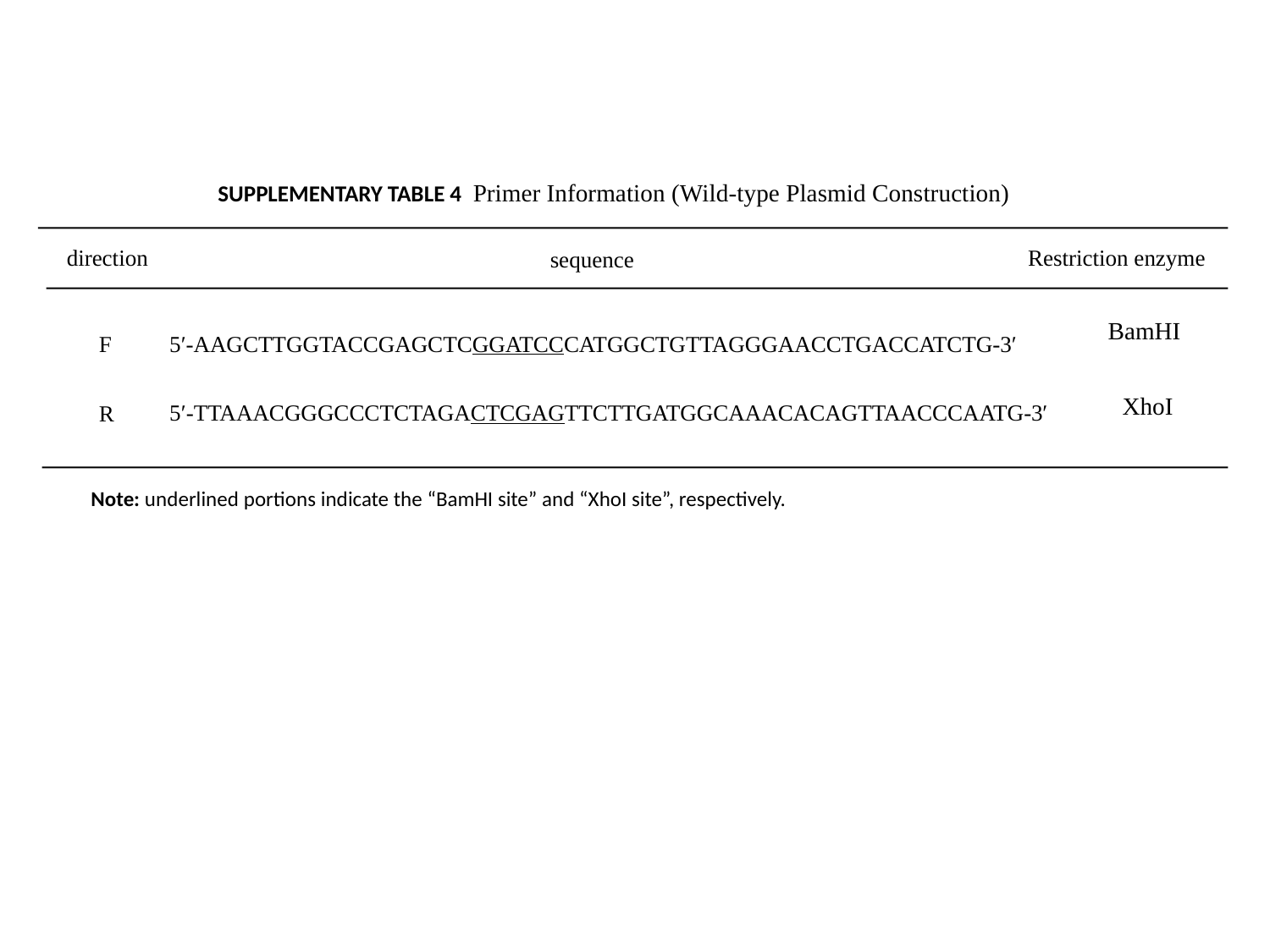

SUPPLEMENTARY TABLE 4 Primer Information (Wild-type Plasmid Construction)
direction
Restriction enzyme
sequence
BamHI
5′‑AAGCTTGGTACCGAGCTCGGATCCCATGGCTGTTAGGGAACCTGACCATCTG‑3′
F
XhoI
5′‑TTAAACGGGCCCTCTAGACTCGAGTTCTTGATGGCAAACACAGTTAACCCAATG‑3′
R
Note: underlined portions indicate the “BamHI site” and “XhoI site”, respectively.
