# Supplementary material for: A novel ABO splice site variant underlying the A3 phenotype: immunogenetic basis and functional dissection
Source: Front Genet. 2026 Jun 19;17:1839848. doi: 10.3389/fgene.2026.1839848 (PMC13327653; doi:10.3389/fgene.2026.1839848)
Supplement: Supplementary file 10 [file Presentation6.ppt]

## Slide 1
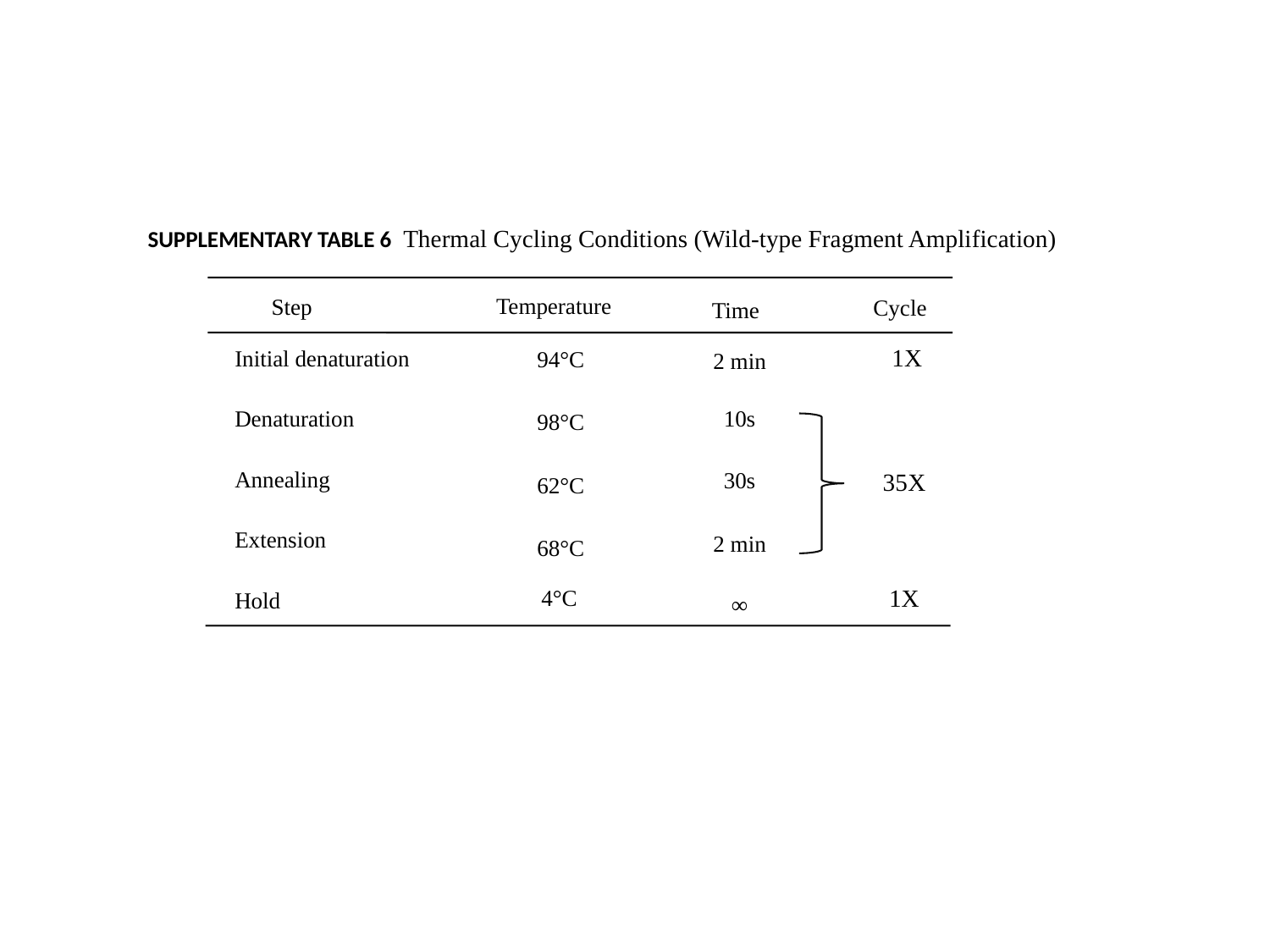

SUPPLEMENTARY TABLE 6 Thermal Cycling Conditions (Wild-type Fragment Amplification)
Temperature
Step
Cycle
Time
1X
Initial denaturation
94°C
2 min
10s
Denaturation
98°C
30s
Annealing
35X
62°C
2 min
Extension
68°C
4°C
1X
Hold
∞
