# Supplementary material for: A novel ABO splice site variant underlying the A3 phenotype: immunogenetic basis and functional dissection
Source: Front Genet. 2026 Jun 19;17:1839848. doi: 10.3389/fgene.2026.1839848 (PMC13327653; doi:10.3389/fgene.2026.1839848)
Supplement: Supplementary file 11 [file Presentation7.ppt]

## Slide 1
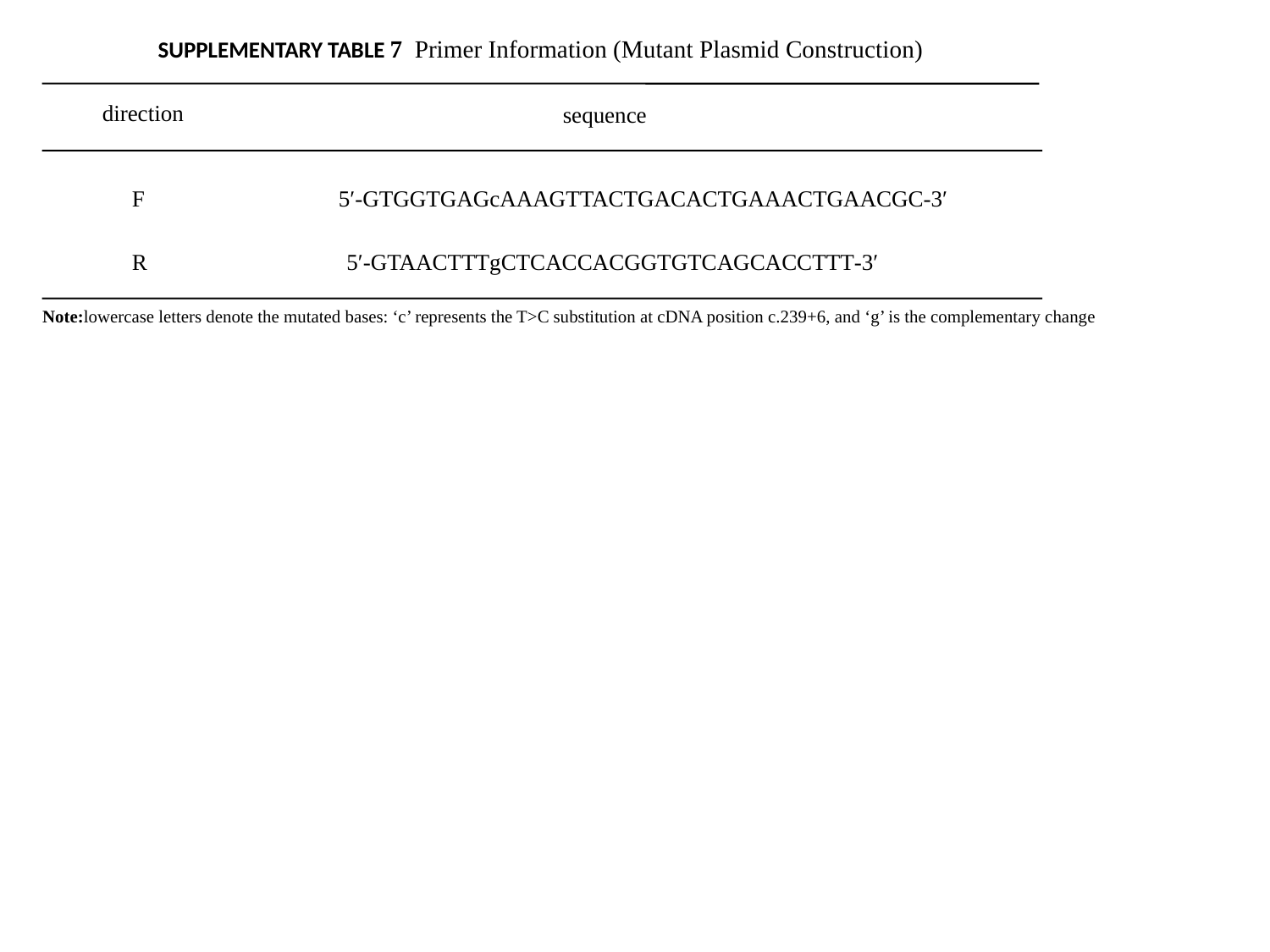

SUPPLEMENTARY TABLE 7 Primer Information (Mutant Plasmid Construction)
direction
sequence
5′‑GTGGTGAGcAAAGTTACTGACACTGAAACTGAACGC‑3′
F
 5′‑GTAACTTTgCTCACCACGGTGTCAGCACCTTT‑3′
R
Note:lowercase letters denote the mutated bases: ‘c’ represents the T>C substitution at cDNA position c.239+6, and ‘g’ is the complementary change
