# Supplementary material for: A novel ABO splice site variant underlying the A3 phenotype: immunogenetic basis and functional dissection
Source: Front Genet. 2026 Jun 19;17:1839848. doi: 10.3389/fgene.2026.1839848 (PMC13327653; doi:10.3389/fgene.2026.1839848)
Supplement: Supplementary file 12 [file Presentation8.ppt]

## Slide 1
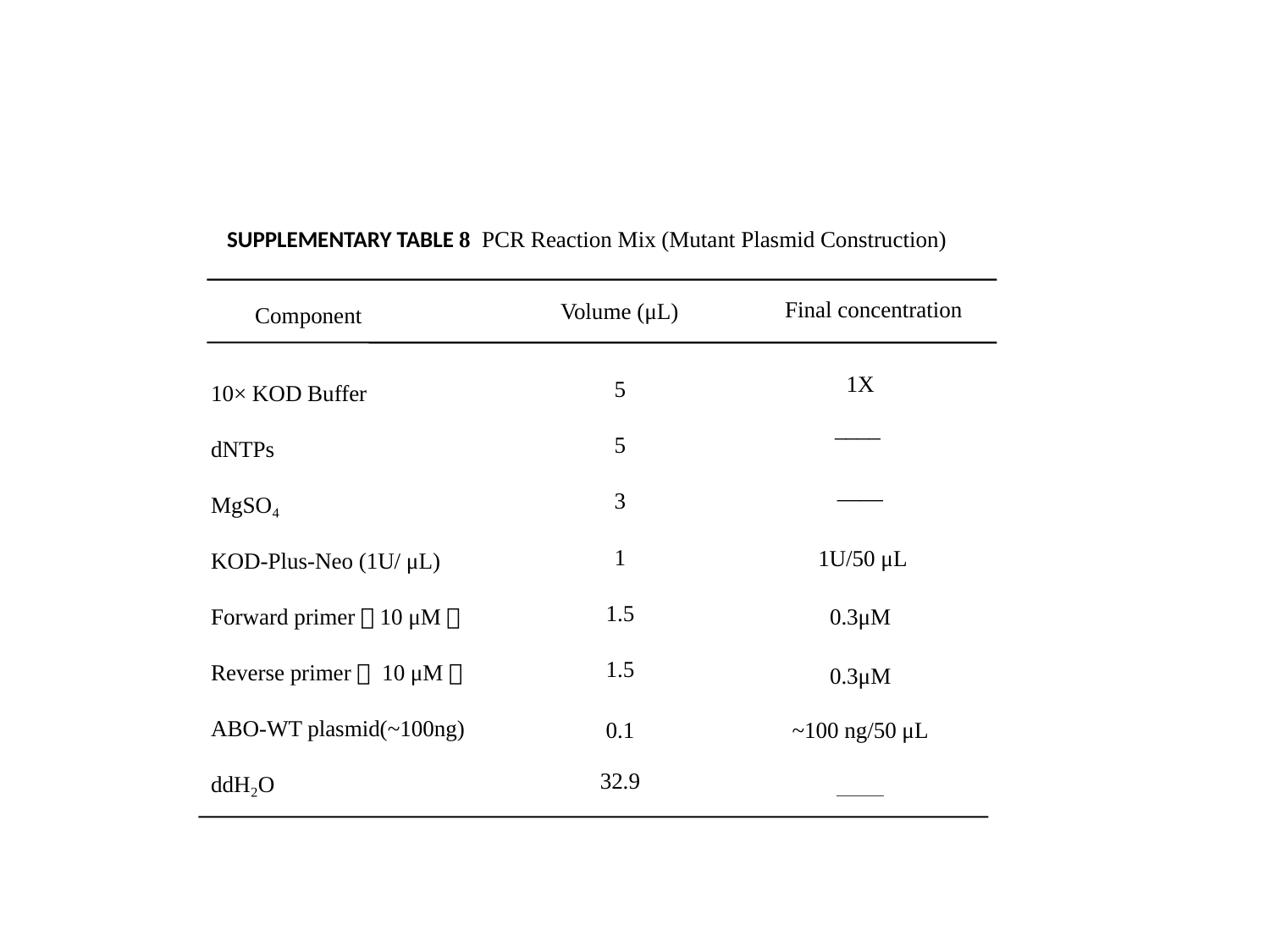

SUPPLEMENTARY TABLE 8 PCR Reaction Mix (Mutant Plasmid Construction)
Final concentration
Volume (μL)
Component
1X
10× KOD Buffer
5
____
dNTPs
5
——
MgSO₄
3
1U/50 μL
KOD-Plus-Neo (1U/ μL)
1
0.3μM
Forward primer（10 μM）
1.5
0.3μM
Reverse primer（ 10 μM）
1.5
ABO-WT plasmid(~100ng)
~100 ng/50 μL
0.1
ddH₂O
_______
32.9
