# Supplementary material for: A novel ABO splice site variant underlying the A3 phenotype: immunogenetic basis and functional dissection
Source: Front Genet. 2026 Jun 19;17:1839848. doi: 10.3389/fgene.2026.1839848 (PMC13327653; doi:10.3389/fgene.2026.1839848)
Supplement: Supplementary file 13 [file Presentation5.ppt]

## Slide 1
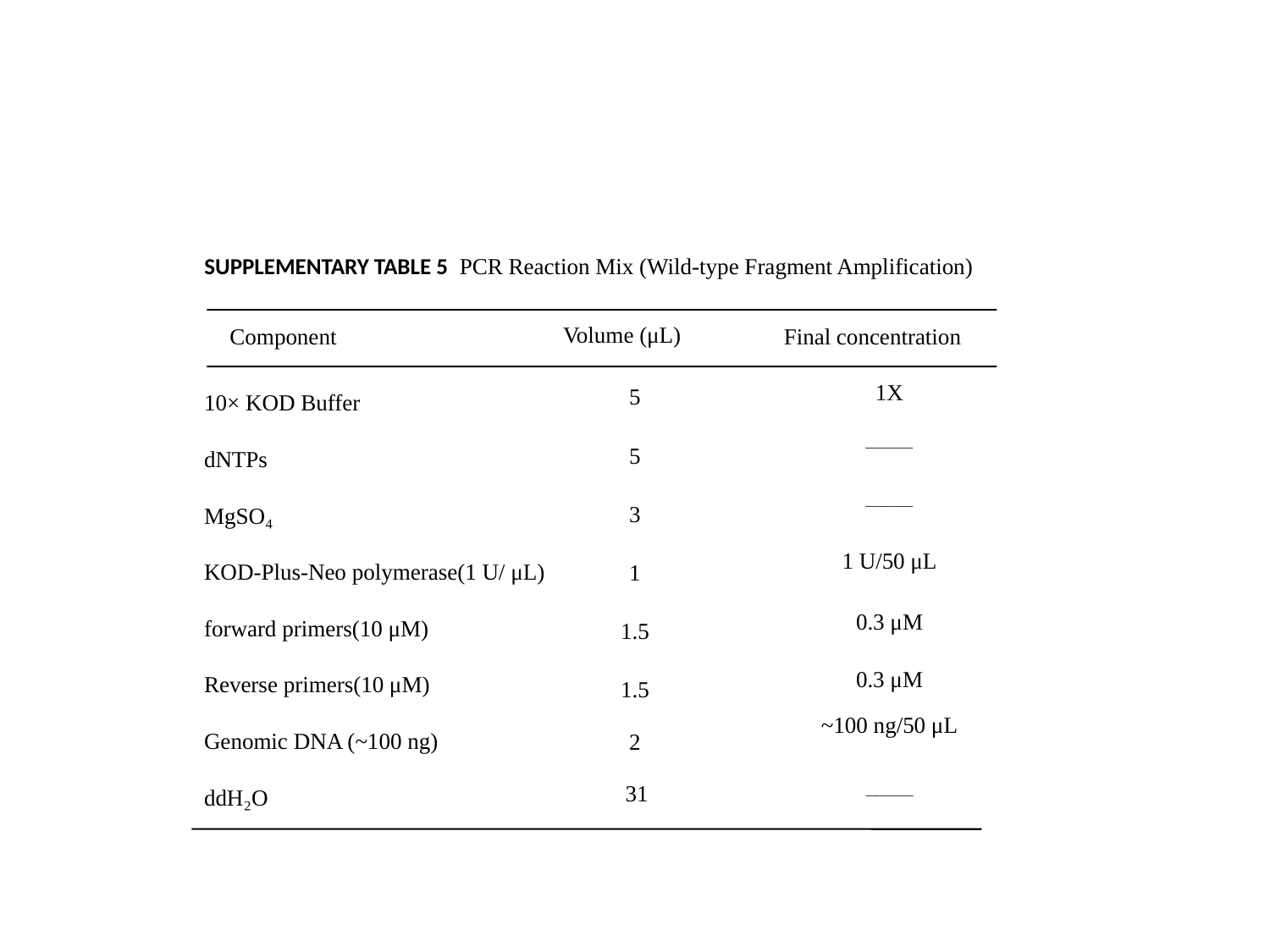

SUPPLEMENTARY TABLE 5 PCR Reaction Mix (Wild-type Fragment Amplification)
Volume (μL)
Component
Final concentration
1X
5
10× KOD Buffer
_______
5
dNTPs
_______
3
MgSO₄
1 U/50 μL
KOD-Plus-Neo polymerase(1 U/ μL)
1
0.3 μM
forward primers(10 μM)
1.5
0.3 μM
Reverse primers(10 μM)
1.5
~100 ng/50 μL
Genomic DNA (~100 ng)
2
_______
31
ddH₂O
